# Production of Xylanase from *Arthrobacter* sp. MTCC 6915 Using Saw Dust As Substrate under Solid State Fermentation

**DOI:** 10.4061/2011/696942

**Published:** 2011-08-11

**Authors:** Sevanan Murugan, Donna Arnold, Uma Devi Pongiya, P. M. Narayanan

**Affiliations:** Microbiology Laboratory, School of Biotechnology and Health Sciences, Karunya University, Karunya Nagar, 641114, Coimbatore, India

## Abstract

Saw dust was used as substrate for xylanase production from *Arthrobacter* sp. MTCC 6915. The study of period of incubation, temperature, pH, carbon, and nitrogen sources for xylanase production was optimized. Xylanase production was found to be optimum at an incubation period of 96 hrs (117.0 U/mL), temperature 30°C (105.0 U/mL), and pH 9.0 (102.9 U/mL). The results showed that the xylanase production was found to be higher in the presence of carboxymethylcellulose (176.4 U/mL) and dextrose (126.0 U/mL). It was also observed that peptone (170.1 U/mL) and beef extract (161.7 U/mL) supported maximum xylanase production.The enzyme was characterized and found to be fairly active at pH 9 (764.4 U/mL) and temperature 60°C (819 U/mL). Even in the present study, a major difference in the production temperature (30°C) and optimal temperature (60°C) of the enzyme activity was observed. However, the pH of the production media and the enzyme activity were found to be the same (pH 9).

## 1. Introduction

Xylanases (endo-1,4-*β*-D-xylan xylanohydrolase) a group of hemicellulolytic enzymes are required for the hydrolysis of *β*-1,4 xylans present in lignocellulosic materials [1]. Xylanase enzymes are extracellular enzymes produced by microorganisms [2], and recently, they have aroused great interest due to their potential application in many industrial processes. The potential applications of xylanase include bleaching noncotton cellulosic fibres, melting hemp and linen fibres, clarifying fruit juice and wines [3] and also increasing the brightness of pulp, breaking up the dough, production of hydrolysates from agro industrial wastes [1], and improvement of nutritional properties in lignocellulosic feed stuff [4]. 

Most studies on the production of xylanases have been performed in submerged liquid culture, and only few works have been reported on xylanase production in SSF using lignocellulosic wastes [5–7]. A number of different sources, including bacteria [8], actinomycetes [9], and yeast [10], have been reported for xylanase production under solid state fermentation (SSF). The most commonly used partial purification of the xylanase enzyme was done using ammonium sulphate precipitation followed by dialysis, since it is highly soluble and nontoxic to proteins and low priced [11]. Chivero et al. [12] and Somogyi [13] used ion exchange chromatography for the purification of xylanase enzyme. The high cost of production of the enzymes has hindered the industrial applications of bioconversion of xylan. It has been reported that several *Arthrobacter* sp. can reduce hexavalent chromium [14], and they are also known to degrade agricultural pesticides and also agricultural wastes [15]. Lignocellulosic substrates being cheap and readily available have now gained considerable interest because of their possible use in secondary fermentation process. Various lignocellulosic substrates involves agricultural waste materials like corn meal, corn cob, wheat bran, rice husk, rice straw, and bagassae and other lignocellulosic materials like sawdust, vegetable garbage, and cellulose can be used as solid state substrates for the production of xylanase enzyme [16]. The purpose of this work was to produce xylanase from *Arthrobacter *sp MTCC 6915 under solid-state fermentation using saw dust as substrate and also to study the influence of conditions of fermentation in enhancing the production of the enzyme and the effect of metal ion concentration on xylanase and also to determine the effect of pH and temperature on the catalytic activity of partially purified xylanase.

## 2. Materials and Methods

### 2.1. Microorganism

*Arthrobacter *sp MTCC 6915 was obtained from Institute of Microbial Technology (IMTECH), Chandigarh, India. The culture was stored at 4°C. It was sub cultured at four week intervals.

### 2.2. Inoculum and Substrate Preparation

100 mL of 2% nutrient broth solution (Hi-Media, Mumbai) was sterilized at 121°C for 15 min, cooled, and inoculated under aseptic condition with *Arthrobacter *sp MTCC 6915 from nutrient agar slant which was stored at 4°C. The broth culture was incubated for 24 hours at ambient temperature.

Saw dust was obtained from the timber industries in Coimbatore, South India. The substrate saw dust was then washed 2-3 times with distilled water and then boiled with distilled water for about 15 minutes. The substrate was dried in an oven and powdered. The powder was sieved using a 40 micron mesh and stored at −20°C until used.

### 2.3. Enzyme Production in Solid-State Fermentation (SSF)

Erlenmeyer flask (250 mL) containing 5 g of sawdust was added to 15 mL mineral basal salt solution (MBSS) medium with the substrate to moisture ratio 1 : 3. The production media was prepared with the following composition (g/L) NaCl, 30.0; KCl, 0.75; MgSO_4_, 7.00; NH_4_Cl, 0.5; K_2_HPO_4_  (10%), 7.00 mL; KH_2_PO_4_  (10%), 3.00 mL and trace metal solution (mg/L): H_3_BO_3,_ 2.83; MnCl_2_·7H_2_O, 1.80; FeSO_4_·7H_2_O, 2.49; Na- K-Tartarate, 1.77; CuCl_2_, 0.33; ZnCl_2_, 0.02; CoCl_2_, 0.04 and Na_2_MoO_4_·2H_2_O, 0.02. The medium and trace metal solutions were autoclaved separately. The flasks were cooled down to room temperature, and a known amount of sterilized trace elements were added. The flask was inoculated with 1 mL of inoculum and the content of the flasks were mixed thoroughly to ensure uniform distribution of the inoculum and incubated at room temperature for 24 hrs.

### 2.4. Optimization of Process Parameters

The various process parameters that influence the enzyme production during SSF were optimized over a wide range. The strategy adopted for standardization of fermentation parameters was to evaluate the effect of an individual parameter and to incorporate it at standardized level before standardizing the next parameter. The effect of incubation period on enzyme activity was examined at different time intervals namely 24, 48, 72, 96, 120, and 144 hrs, respectively, at ambient temperature (28 ± 2°C) and pH 7.0. The effect of temperature on enzyme activity was examined at 20°C, 25°C, 30°C, 35°C, and 40°C at pH 7.0 for 96 hours (optimal incubation period). Similarly, the effect of pH on enzyme activity was examined at different pH namely 6, 7, 8, 9, and 10, respectively.

The effect of supplementation of additional carbon and nitrogen sources to saw dust was examined using carbon sources consisting of carboxymethylcellulose, dextrose, lactose, and sucrose (1%–5%). Similarly, the nitrogen sources examined were potassium nitrate (KNO_3)_, ammonium sulphate [(NH_4_)_2_SO_4_], beef extract, and peptone (1%–5%). The optimum concentration of the carbon and nitrogen sources was also determined. The synergistic effect of various carbon and nitrogen sources which showed high xylanase activity was also examined to determine the best combination of carbon and nitrogen sources for the xylanase activity. The combined carbon and nitrogen sources include carboxymethylcellulose and peptone, carboxymethylcellulose and beef extract, dextrose and peptone, and dextrose and beef extract. For the same supplementation studies, the enzyme production was carried out at optimum temperature (30°C) and pH 9.0 for 96 hours.

### 2.5. Preparation of Enzymes

Erlenmeyer flask (500 mL) containing 10 g of sawdust and 30 mL of MBSS, pH 9.0 along with the carbon and nitrogen sources required for the production of the enzyme was sterilized, cooled, and inoculated with 1 mL of the pregrown culture of *Arthrobacter *sp MTCC 6915 in nutrient broth. The flasks were incubated at room temperature for 96 hours, and the enzyme was extracted using Glycine-NaOH buffer.

### 2.6. Enzymatic Assay

The xylanase activity was assayed using 1% birchwood xylan as the substrate. 1% xylan was dissolved in 50 mM Glycine-NaOH buffer (pH 9.0). The reaction mixture containing 40 *μ*L of an appropriate dilution of the enzyme and 1 mL of the substrate was incubated for 10 min. The amount of reducing sugars liberated was determined by using 3,5-dinitrosalicylic acid method (DNS method) [17]. The absorbance of the reference samples (substrate solution incubated without enzyme and diluted enzyme solution in buffer) was deduced from the values of the test samples which were measured by monitoring the optical density at 540 nm. The unit of xylanase activity was defined as the number of *μ* moles of reducing sugars formed (measured as xylose) per min under the assay conditions.

(1) $$\begin{array}{cc} & \text{Enzyme Activity}\\ & \;\;=\frac{\text{g}\;\text{of}\;\text{maltose}\;\text{released}}{\text{Volume}\;\text{of}\;\text{enzyme}\;\text{used}\;\times \;\text{Time}\;\text{of}\;\text{assay}}\\ & \;\;\;\times \frac{\text{Total}\;\text{volume}\;\text{of}\;\text{assay}\;\left(\text{mL}\right)}{\text{Volume}\;\text{used}\;\text{in}\;\text{Colorimetric}\;\text{determination}}. \end{array}\;$$

### 2.7. Purification of the Enzyme

#### 2.7.1. Partial Purification by Ammonium Sulphate Precipitation

The contents of the flask were suspended in 100 mL of 50 mM Glycine-NaOH buffer (pH 9) vortexed thoroughly and centrifuged (10,000 rpm for 10 min, 4°C). All proteins are precipitated by ammonium sulphate [18]. About 100 mL of culture filtrate was treated with 52.3 g of ammonium sulphate to give 80% saturation and most of the proteins were precipitated out. This was left overnight, and the precipitate was collected by centrifugation at 10,000 rpm for 10 min [12]. The precipitate was dissolved in phosphate buffer (50 mM, pH 8.0), and the enzyme activity was determined in both the precipitate and supernatant. The sample having maximum enzyme activity was used for further studies.

#### 2.7.2. Dialysis

The precipitate obtained was dissolved in phosphate buffer (50 mM, pH 8.0), and the supernatant was dialysed against the same buffer for 24 hours. Dialysis was carried out using cellulose tubing (molecular weight cutoff 13,000 kDa). The enzyme activity was determined in both the precipitate and supernatant. The sample (precipitate) having maximum enzyme activity was used for further studies [12, 18].

#### 2.7.3. Ion Exchange Chromatography

Concentrated enzyme was loaded onto a anion exchange DEAE Sepharose FF (Sigma-Aldrich Co, USA) column (15 mm diameter and height 100 mm) at a flow rate of 0.5 mL/min. Equilibration and elution were performed first with 0.05 M Na-Phosphate buffer to remove unbound proteins and then with a linear salt gradient from 0 to 3 M NaCl. Fractions were collected and analysed for xylanase activity and protein content. Active fractions were pooled and concentrated and then purified using cation exchange CM Sepharose FF (Sigma-Aldrich Co, USA) column. The active fractions were pooled and used for further studies [11].

### 2.8. Enzyme Characterization-Temperature and pH Profile

The optimal temperature for the purified xylanase was obtained by assaying the enzyme activity at different temperatures 50°C, 60°C, 70°C, 80°C, 90°C, and 100°C at pH 9.0. At each temperature, the enzyme 40 *μ*L along with 1 mL of substrate (birchwood xylan) was incubated for 40 mins. The residual activity of the enzyme was measured at 540 nm. Similarly, the relative xylanase activity using 1% (w/v) birchwood xylan was determined at various pH levels. The pH range used varied from 4 to 11. Three different buffers (0.05 M) were used. Citrate buffer was used for pH 4 to 6, Phosphate buffer was used for pH 7 and 8, and glycine- NaOH buffer was used for pH 9 to 11. The enzyme, along with the substrate and the respective buffers, was incubated for 60°C at 3 hours. The residual activity of the enzyme was measured at 540 nm.

### 2.9. Effect of Metal Ion Concentration

Enzyme was incubated with 1 mM solution of the following metal salts such as CaCl_2_, MgCl_2_, MgSO_4_, HgCl_2_, FeSO_4_, CuCl_2_, and ZnCl_2_ and metal chelator such as EDTA for 1 hour at room temperature (28 ± 2°C). The residual activity of the enzyme was measured at 540 nm.

## 3. Results and Discussion

### 3.1. Optimization of Process Parameters

**Figure 1:**
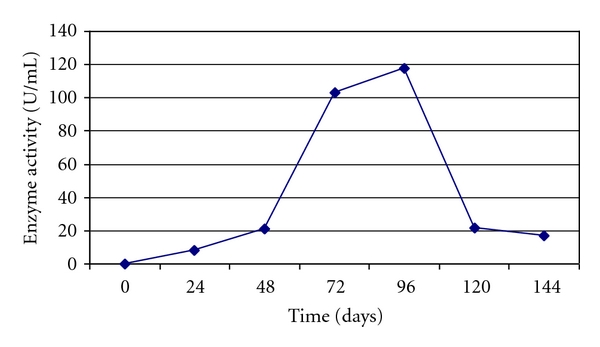
Effect of incubation period on xylanase activity of *Arthrobacter *sp MTCC 6915 using saw dust as substrate.

The substrate used in the present study was saw dust, and when grown on saw dust, the maximum enzyme production observed was at 96 hrs (117.6 U/mL) for every 24 hours at 540 nm. Only small quantities of xylanase were produced during first 24 hrs (8.4 U/mL) of fermentation but xylanase production rapidly increased between 48 and 96 hrs (Figure 1). Whereas *Aspergillus niger* when grown on rice straw under solid state fermentation showed maximum xylanase production after 120 hrs of incubation [19]. *Penicillium oxalicum* when grown on wheat bran under solid state fermentation, showed maximum production after 144 hrs [20].

**Figure 2:**
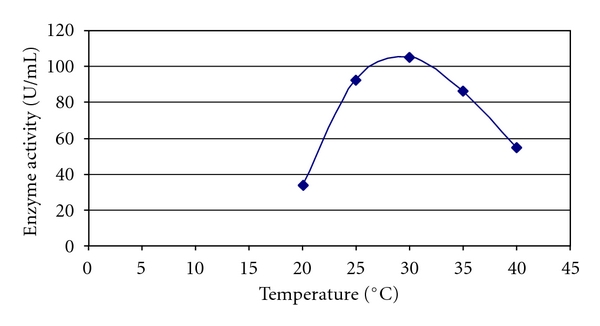
Effect of temperature on xylanase activity of *Arthrobacter *sp MTCC 6915 using saw dust as substrate.

The level of increasing xylanase activity (105.0 U/mL) was found to be optimum at 30°C. However, increase in temperature beyond 30°C led to a decline in the production of enzyme. At a temperature of 25°C (92.4 U/mL), the enzyme activity was found to be little lower than that at 30°C. The minimum activity was, however, found to be at 20°C (33.6 U/mL) (Figure 2). Similar results were observed, when *Arthrobacter *sp MTCC 5214 was grown on wheat bran [18]. 

**Figure 3:**
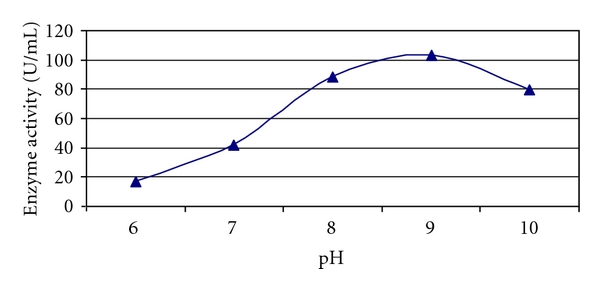
Effect of pH on xylanase activity of *Arthrobacter *sp. MTCC 6915 using saw dust as substrate.

The highest enzyme activity was found to be optimum at pH 9 (102.9 U/mL) and minimum at pH 6 (16.8 U/mL) (Figure 3). Xylanase at pH value 7–9 makes it suitable for bioleaching applications. Among the xylanases from *Bacillus *sp, highest pH optima had been reported for *Bacillus *Tar-1, C-125 and *Bacillus *sp. NCL-86-6-10 [21, 22]. However, several alkaline-tolerant fungal xylanases have also been characterized [23–25]. Thus, the results of the current study are in accordance with the previous reports. 

**Figure 4:**
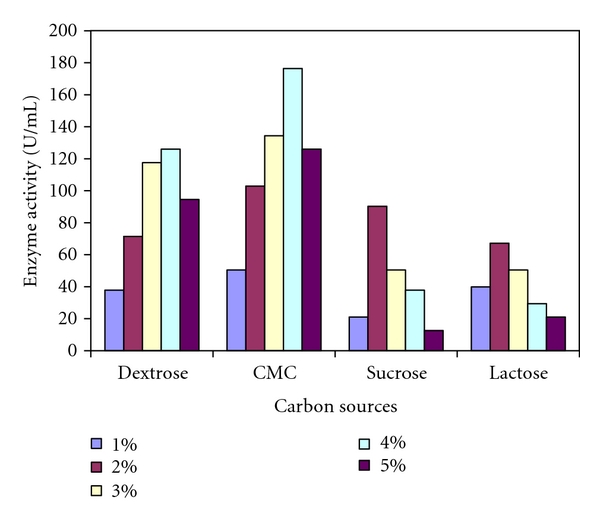
Effect of carbon sources on xylanase activity of *Arthrobacter *sp. MTCC 6915 using saw dust as substrate.

The impact of supplementation of external carbon source at 1%–5% concentration on xylanase activity was studied, in which four different carbon sources were examined and the results are shown in Figure 4. All the carbon sources decreased the xylanase activity at 5%. The decreases may also be caused by the complex carbon sources present in the production media. On the basis of analyzing the results, it may be concluded that the microorganisms necessitate a low level of carbon in order to produce enzymes, because they are the limiting factor. Among the four sources selected, 4% of carboxymethylcellulose (176.4 U/mL) was found to exhibit the maximum enzymatic activity. Similarly, 4% of dextrose (126.0 U/mL) also showed relatively higher, however, found lower than that of carboxymethylcellulose. Sucrose and lactose showed very low enzymatic activity. In the present study, it was found that 4% carboxymethylcellulose and dextrose are the good sources for the production of xylanase enzyme (Figure 4). Effect of carbon sources on the production of xylanase by *P. oxalicum* showed higher enzyme activity for carboxymethylcellulose, when compared to sucrose, lactose, and glucose [20]. Thus, the supplementation of production media with external carbon source had positive effect on enzyme production by *Arthrobacter *sp MTCC 6915. This shows that the findings of the current studies are in accordance with the earlier reports. 

**Figure 5:**
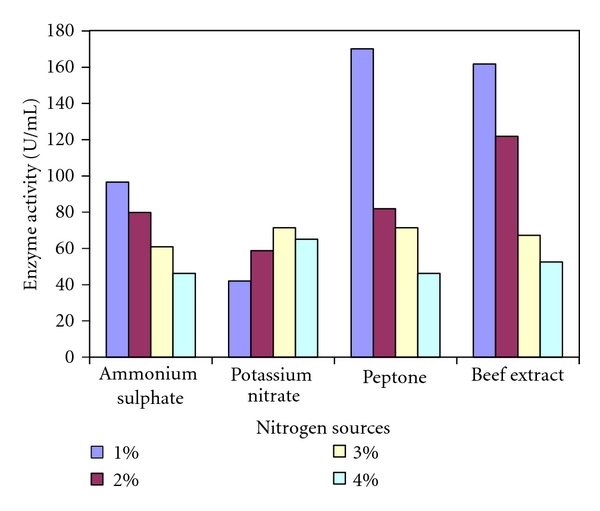
Effect of nitrogen sources on xylanase activity of *Arthrobacter *sp. MTCC 6915 using saw dust as substrate.

Among the four nitrogen sources tested 1% peptone (170.1 U/mL) was found to exhibit the maximum enzymatic activity (Figure 5). Similarly, 1% beef extract (161.7 U/mL) also showed relatively higher activity, however, lower than that of peptone. Ammonium sulphate and ammonium nitrate showed very low enzymatic activity. In the present study, it was found that 1% of peptone and beef extract are good supplements for the production of xylanase enzyme (Figure 5). *Enterobacter *sp MTCC 5112 showed highest xylanase production when the growth medium was supplemented with peptone [18]. Effect of nitrogen sources on production of xylanase by *Penicillium oxalicum* showed higher enzyme activity for peptone when compared to beef extract, ammonium sulphate, and ammonium nitrate [20]. Thus results of the present study are similar to the earlier findings.

### 3.2. Synergistic Effects of Various Carbon and Nitrogen Sources

Four different combinations of two carbon (carboxymethylcellulose and dextrose) and nitrogen sources (peptone and beef extract) were taken to determine which combination exhibited the highest enzyme activity. The two carbon sources which exhibited the highest level were 4% each of carboxymethylcellulose (176.4 U/mL) and dextrose (126.0 U/mL) and nitrogen sources 1% each of peptone (170.1 U/mL) and beef extract (161.7 U/mL). It was found that the combination of 4% dextrose and 1% peptone exhibited the maximum enzymatic activity (102.9 U/mL).

### 3.3. Purification of the Enzyme

The enzyme was isolated, purified, and characterized at room temperature (28 ± 2°C). After the partial purification of the enzyme by ammonium sulphate precipitation, the precipitate was separated by centrifugation, then the supernatants and precipitates were subjected to a cellulose tubing bag for dialysis. After the dialysis, enzyme assay was carried out for both the supernatant and precipitate and found that the precipitate (453.6 U/mL) showed the maximum enzymatic activity which indicates that the enzyme was present in the precipitate. For further purification of the enzyme, ion-exchange chromatography was performed [26], and it was found that the precipitate (804.0 U/mL) retained maximum enzyme activity than compared to the supernatant.

### 3.4. Enzyme Characterization: Temperature and pH Profile

**Figure 6:**
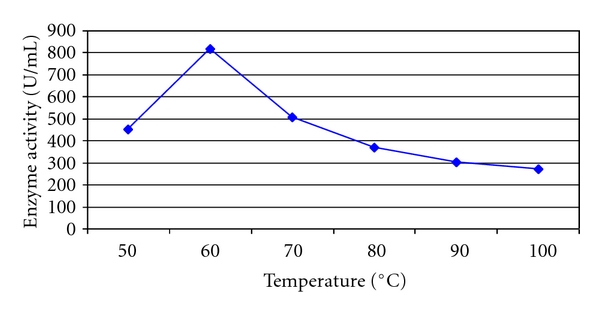
Temperature profile of xylanase activity of *Arthrobacter *sp. MTCC 6915 using saw dust as substrate.

The maximum enzymatic activity was observed at pH 9.0 (764.4 U/mL) after 3 hours of incubation. The enzyme activity was considerably higher at pH 10.0 (739.2 U/mL) but comparatively lesser than at pH 9.0. The enzyme activity decreased greatly and lost most of its activity in acidic pH range after 3 hours (Figure 6). There are only few bacterial xylanases reported till date with pH optima of >9.0 when grown on solid-state fermentation. Xylanase isolated from *Bacillus subtilis* which was grown on solid-state fermentation using oat spelt xylan showed maximum activity at pH 6.0 [27]. Xylanase isolated from *B. licheniformis* A99 [28] had the optimal pH of 7.0. Similarly, xylanase isolated from *B. coagulans* BL69 grown on soybean residue [29] showed an optimum pH of 7.0. When *Arthrobacter *sp MTCC 5214 grown on wheat bran showed optimum at pH 9.0 [18]. In view of these observations, the properties of the xylanase produced by *Arthrobacter *sp MTCC 6915 are much more superior to those reported for xylanases isolated from other bacteria. 

**Figure 7:**
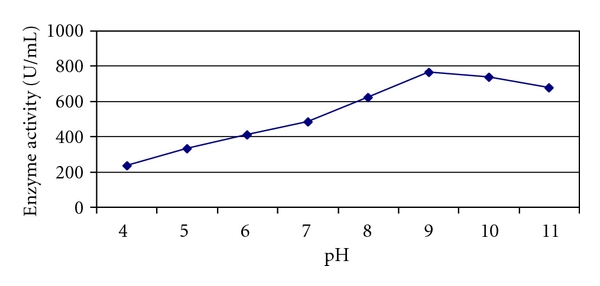
pH profile of xylanase activity of *Arthrobacter *sp. MTCC 6915 using saw dust as substrate.

Maximum enzymatic activity was observed at temperature of 60°C (819.0 U/mL) after 40 min incubation. The enzyme activity was considerably higher at 50°C (453 U/mL) and 70°C (508.2 U/mL) though comparatively lesser than 60°C (Figure 7). Xylanase isolated from *B. coagulans* BL69 grown on soybean residue [29] showed activity over a wide range of temperatures (45°C –75°C). *B. subtilis* produced a xylanase enzyme, which had a highest activity at 60°C temperature, when grown on oat spelt xylan under solid-state fermentation [27]. Similarly, xylanase obtained from *Streptomyces actuosus* A-151 grown on rice bran showed optimum temperature of 60°C –70°C [30]. *Bacillus* sp. JB-99 grown on rice bran in solid-state fermentation produced xylanase with optimum activity at 50°C [31].

### 3.5. Effect of Metal Ion Concentration

Xylanase activity was assayed in the presence and absence of metal ions, and a metal chelator EDTA. Both Hg^2+^(25.2 U/mL) and EDTA (10.5 U/mL) inhibited the activity of the xylanase enzyme. The inhibition of the enzyme by Hg^2+^ ions may be due to its interaction with sulphydryl groups, suggesting that there is an important cysteine residue in or close to the active site of the enzyme. The inhibition of the xylanase activity in the presence of EDTA suggests that metals are needed for the enzymatic reaction. The xylanase activity was greatly elevated by the addition of Zn^2+^, Fe^2+^, Cu^2+^, Mg^2+^, and Ca^2+^ ions (Table 1). It is not certain from the above studies, whether these ions/agents could be binding to the enzyme, causing conformational changes that resulted in increased enzyme activity, or the xylanase requires a metal ion on the active site. Nakamura et al. [32] showed that Hg^2+^ ions inhibited the enzyme activity. Cesar and Mrša [33] for *T. lanuginosus* showed that Ca^2+^ enhanced xylanase activity. Mn^2+^ and Zn^2+^ ions also had positive effect on xylanase activity [34]. Ghanem et al. [35] for *Aspergillus terreus* showed that Ca^2+^ enhanced xylanase activity. Khandeparkar and Bhosle [18] for *Enterobacter *sp. showed that EDTA inhibited the enzyme activity. Similar results were observed in the present study.

## 4. Conclusion

The results strongly indicate that the SSF system using saw dust as substrate is an economical method for the production of xylanase at extremely low operational cost, as cheap and abundant wastes are generated in the timber industries. *Arthrobacter *sp MTCC 6915 used in the present study is novel, and there are no published reports on the production of xylanase by this culture. However, the amount of enzyme produced by the *Arthrobacter *sp MTCC 6915 differed with each of the carbon and nitrogen sources. For the efficient solid waste management, this waste can be used at the maximum, as continuous accumulation of industrial waste possess a serious environmental hazard. The xylanase enzyme produced by the *Arthrobacter *sp MTCC 6915 is thermostable and alkalophilic which has wide industrial applications. The future prospects of this study is that a better thermostable and alkalophilic enzyme can be produced which can be used for industrial applications so that the enzyme can withstand the temperature and the alkaline pH.

## Figures and Tables

**Table 1:** Effect of Metal Ion Concentration on Xylanase Activity of *Arthrobacter *sp MTCC 6915 using saw dust as substrate.

Metal salt solution	Enzyme activity (U/mL) at 540 nm
ZnCl_2_	168
FeSO_4_	142.8
MgSO_4_	157.6
MgCl_2_	155.4
CuCl_2_	142.8
HgCl_2_	25.2
CaCl_2_	130.2
EDTA	10.5

ZnCl_2_—zinc chloride; CuCl_2_—copper chloride; FeSO_4_—ferrous sulphate; HgCl_2_—mercuric chloride; MgSO_4_—magnesium sulphate; CaCl_2_—calcium chloride; EDTA—ethylene diamine tetra acetic acid; MgCl_2_—magnesium chloride.
